# Developing a prioritisation framework in an English Primary Care Trust

**DOI:** 10.1186/1478-7547-4-3

**Published:** 2006-02-17

**Authors:** Edward CF Wilson, John Rees, Richard J Fordham

**Affiliations:** 1 UEA/NHS Health Economics Support Programme, Health Economics Group, School of Medicine, Health Policy & Practice, University of East Anglia, Norwich, NR4 7TJ, UK; 2 West Norfolk Primary Care Trust, St James, Exton's Road, King's Lynn, Norfolk, PE30 5NU, UK

## Abstract

**Background:**

In the English NHS, Primary Care Trusts (PCTs) are required to commission health services, to maximise the well-being of the population, subject to the available budget. There are numerous techniques employed to make decisions, some more rational and transparent than others. A weighted benefit score can be used to rank options but this does not take into account value for money from investments.

**Methods:**

We developed a weighted benefit score framework for use in an English PCT which ranked options in order of 'cost-value' or 'cost per point of benefit'. Our method differs from existing techniques by explicitly combining cost and a composite weighted benefit score into the cost-value ratio.

**Results:**

The technique proved readily workable, and was able to accommodate a wide variety of data and competing criteria. Participants felt able to assign scores to proposed services, and generate a ranked list, which provides a solid starting point for the PCT Board to discuss and make funding decisions. Limitations included potential for criteria to be neither exhaustive nor mutually exclusive and the lack of an interval property in the benefit score limiting the usefulness of a cost-value ratio.

**Conclusion:**

A technical approach to decision making is insufficient for making prioritisation decisions, however our technique provides a very valuable, structured and informed starting point for PCT decision making.

## Background

The demand for health care will always exceed the capacity of the available resources. Therefore decisions must be made as to which treatments and services to commission (purchase), in order to maximise the well-being of the population, subject to the available budget.

The objective of the English National Health Service (NHS) is not explicitly defined. However, the role of the Department of Health is to "improve the health and wellbeing of people in England" [1]. Primary Care Trusts (PCTs) are responsible for commissioning an appropriate basket of health care services to achieve this in their local population (approximately 100,000 – 200,000). PCTs can commission from a variety of care providers including NHS and private sector hospitals and clinics, the voluntary sector and (in some cases) social services.

A PCT in the East of England previously made these decisions by means of a 'cluster group' consisting largely of chief executives, finance directors, and occasional clinical input from both the PCT and the local major acute trust (hospital). Both the PCT and the acute trust possessed a list of desired purchases (e.g. new staff and new equipment from the acute trust, and a mixture of health care packages from the PCT), and agreement was reached as to what was and would not be purchased through an informal process of arbitrage.

The key advantage of this system was that it was relatively quick and cheap to operate. However mechanisms such as this and others (for example needs assessment and 'historical allocation') suffer flaws as they are 'non-economic'. That is, either they do not consider the relative health gain from alternative courses of action (the opportunity cost) or do not seek to optimise the well-being for the population for the given budget (or both) [2]. The consequence of either of these tools is inefficient allocation of scarce resources, leading to a potential loss of well-being to the population. Furthermore, the existing approach was supplier rather than commissioner led, almost entirely input-focussed with no consideration of outcomes, and little or no evidence used to support the bids. The actual decision making process was somewhat opaque making it difficult to justify why and in whose interests a particular decision was made.

The PCT Director of Public Health (JR) suggested initially to the authors a basic framework incorporating epidemiological, quality of life and "intervention" elements in a matrix against which disease based areas could be tested. The authors were consulted and requested by the PCT to expand this basic framework and devise a tool that would enable the "fair and equitable" commissioning of new services. The tool would require bids for funds to be outcome based and fully costed, making use of the literature and other evidence in support. Comparison of the relative value for money of bids could then be made, and funds allocated to maximise the benefit ('well-being', health gain and / or other objectives such as equity) for the monies available.

The assumed scenario was that a certain pool of growth monies would be available from which competing bids could be funded in ranked order. The tool would allow a rational and defensible decision to be made as to which to prioritise.

In this paper we describe the development of such a tool, and discuss a number of its strengths and weaknesses, including the results of some early testing.

## Method

The method adopted in the study PCT is based on a multi-criteria analysis approach. This generates a weighted benefit score (WBS) which is combined with cost to generate a cost-value ratio. A low cost-value ratio implies a better value for money programme than one with a higher ratio. There are seven steps:

1. Determine the benefit criteria

2. Weight the criteria

3. Score each programme against each criterion

4. Calculate weighted benefit score

5. Combine with cost data to generate cost-value ratio

6. Rank in order of cost-value ratio

7. Discussion of results.

### 1. Determine the benefit criteria

This is done at a brainstorming session, where 'desired' characteristics of a health care programme are identified. The criteria must, as far as is possible, be exhaustive (to avoid underestimation of benefit), and mutually exclusive (to avoid double counting). These criteria are essentially an explicit definition of the aims of the PCT (and therefore should be consistent with the aims of the NHS as a whole).

### 2. Weight the criteria

Some criteria may be considered more important than others. Once the list of criteria has been determined, it is necessary to weight them relative to one another. This is done by allocating 100 percentage points amongst the criteria.

Stages 1 and 2 define the benefits valuation framework. The remaining stages relate to scoring individual programmes against the framework.

### 3. Score each programme against each criterion

Each programme submitted to this process is scored against each criterion independently, on a scale of 0–10. A score of 5 indicated no change compared with current service provision in a particular criterion. A score greater than this implied an improvement, and below, a deterioration. A scale of 0–10 was chosen as it was hoped this would allow enough scope for scorers to discriminate between programmes without spurious precision, and 5 chosen to represent 'current performance' to accommodate programmes benefiting some criteria, but at the expense of performance elsewhere (for example, improvement in access and equity at the expense of some effectiveness).

### 4. Calculate weighted benefit score

For each programme, the score for each criterion is multiplied by the weight on that criterion. The weighted scores are added up across all criteria to obtain a weighted benefit score for the entire programme.

### 5. Combine with cost data to generate cost-value ratio

The net financial impact (total cost of the proposal less any savings from discontinued services and avoided NHS activity) is divided by the weighted benefit score to generate the cost-value ratio.

### 6. Rank in order of cost-value

Programmes at the top of the list have the lowest cost-value ratio. This means that they achieve the criteria more efficiently that those with a higher cost-value ratio, and therefore represent better value for money. Allocating resources from the top of the list downwards then ensures that those programmes achieving the stated aims of the PCT most efficiently are prioritised above less efficient ones.

### 7. Discussion of results

The ranked list of proposals by cost-value forms a recommendation submitted to the PCT Board. The Board reviews the list and may make changes from the recommendation as it sees fit. This is a critical stage as, with any prioritisation framework, there will be imperfections in the mechanism which may generate apparently perverse results. It is essential, however, that changes to the recommended ranking are not arbitrary and must be justified and documented, thus retaining transparency in the system.

## Results

### Devising the framework

The framework for calculating benefit scores was devised and tested at a workshop held in October 2003. The day was split into three sessions (defining the criteria, weighting them and testing the framework with a number of mock proposals). The test session demonstrated the feasibility of the framework.

**Figure 1:**
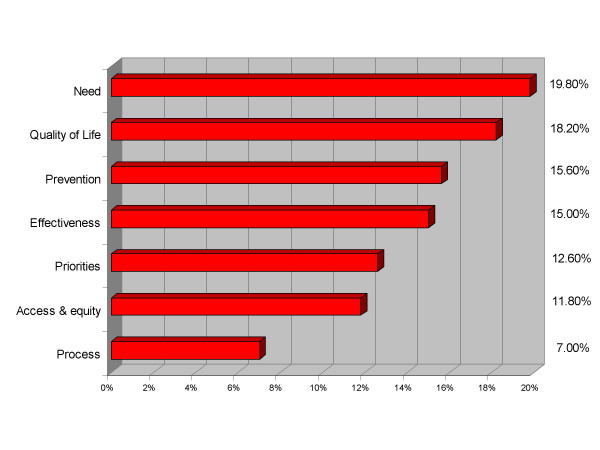
**Criteria and weights**. Mean weightings of the criteria relative to one another.

Around 20 representatives from across the local health economy, including NHS clinicians, PCT and acute trust managers, social services and the voluntary sector attended the workshop, and were split into 5 mixed groups for the day. Sessions 1 and 2 generated the evaluation framework comprising seven criteria (Table 1), each weighted relative to one another (Figure 1).

**Table 1:** Criteria definitions (alphabetically)

**Criterion**	**Definition**
Access & equity	• Does this proposal increase or improve access to services for the target population? • Does this proposal have any impact on access to services for other populations or other NHS agencies (positive or negative)? • Is this a locally based service? • Is this service available to all who need it? • Is this patient-centred healthcare? Do they get a say in the delivery of their care? Is there demonstrable 'patient & public involvement'? • Does the proposal enable treatment in an appropriate environment? • Does the proposal raise the profile of an important but currently low profile disease / condition?
Effectiveness	• Is the proposal proven to work? (what evidence is there for it working?) • What is the quality / grade of the evidence? (e.g. well conducted randomised controlled trial versus expert opinion). • What is the balance of risk and benefit to the patient? • Will the proposal result in enough activity to maintain quality? (clinical governance issues)
Local & National Priorities	• How far towards meeting an explicit national or local target does this proposal go (for example, National Institute for Clinical Excellence, National Service Frameworks, Local Development Plans etc)?
Need	• What is the prevalence / incidence of the disease or condition this proposal is intended to treat? • What is the current mortality or morbidity associated with this disease/condition? (note this should take into account the impact of existing treatments) • Does this proposal meet an identified health need (either local or national)? • Does it meet public expectations / does it meet a local health want?
Prevention	• Does the programme focus or put greater emphasis on prevention of ill health? (For example through health promotion, screening/ immunisation or reduction in future morbidity.)
Process	• Is the proposal achievable within a realistic timescale? • Does the proposal involve multi-agency working / partnership working across different areas of the NHS (and wider bodies)? • Is the proposal acceptable politically?
Quality of life	• What impact does the intervention have on different domains of quality of life (e.g. disability reduction, increase in independence, pain reduction, whether it allows a patient to play active role in society, social relationships, etc)? • What is the potential QALY (Quality Adjusted Life Years) gain from the intervention? • Does the proposal decrease (future) care needs for the patient, carer or family? • What evidence is there for the patient experience / satisfaction?

The weighted criteria can be grouped into four broad 'levels of importance'. Need and quality of life were considered the most important, with just under 20% of the total weighting each. Prevention and effectiveness were considered of similar importance at 15% each. The third grouping consisted of priorities and access & equity issues, and finally process (referring to the feasibility, speed of implementation and integrated working) was considered the least important of the criteria. Each of the five groups weighted the criteria independently and averages were taken to arrive at the percentages. There was some variation between the groups in weighting each criterion and the impact of this is investigated in the sensitivity analysis.

### Testing the framework

The final session of the day was a mock scoring round to test the feasibility of the framework. This comprised four fictitious proposals to be scored against the criteria, and was an important exercise to enable participants to see the complete process. The four proposals comprised an acute service development (W), a preventative programme (X), a screening programme (Y) and a mental health programme (Z).

Ideally, each group would have scored all proposals, but due to time limitations, each group scored only one or two. For each proposal, weighted average scores were calculated from the 5 group scores against each criterion (Table 2).

**Table 2:** Raw scores by programme

	Criteria							Weighted score
	
	Effectiveness	Quality of Life	Access/Equity	Need	Priorities	Prevention	Process	
Weights	0.15	0.182	0.118	0.198	0.126	0.156	0.07	
Programme								
W (acute service)	6	7.6	8.6	6.4	4.8	4.6	4.6	6.21
X (preventative)	2	7	4	5	4	10	5	5.45
Y (screening)	7	7	9	9	9	6	9	7.87
Z (mental health)	1.3	2.3	3.3	2.7	7	3.7	0.7	3.05

The highest scoring programme was proposal Y (screening), followed by W (acute service), X (preventative) and finally Z (mental health). This ranking though only takes into account benefit score, and not the cost or resources required. When the net financial impact is divided by the weighted benefit score (to calculate the cost-value ratio), the ranking changes to Y, X, W, Z (Table 3).

**Table 3:** Cost-value ratio ranking

Bid	Net Cost Impact	Score	Cost/Point
Y (screening)	-£7,780,000	7.87	-£988,564
X (preventative)	£21,600	5.45	£3,693
W (acute service)	£100,000	6.21	£16.585
Z (mental health)	£217,000	3.05	£71,225

Bid Y was ranked first, as not only was this the highest scoring programme, but it also resulted in a net cost saving, therefore representing a good investment. Bids X, W and Z followed in order of cost per point. This list would be the recommended funding order for the programmes, commencing with bid Y, then X and so on until all funds had been allocated.

### Sensitivity analysis

**Figure 2:**
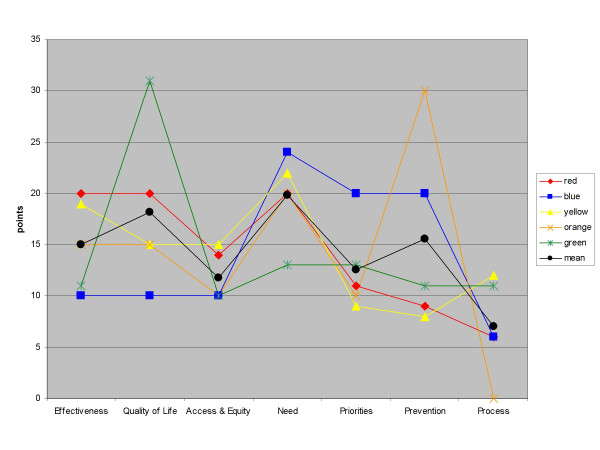
**Variance in criteria weights**. Variation in criteria weightings between groups.

As only one or two groups scored each proposal, it was not possible to analyse any variance in proposal scores. However, each group did weight the criteria independently. Most of the groups were broadly consistent with each other in their criteria weightings, with least variation around the 'access and equity' and 'need' criteria (Figure 2). However, there are a couple of exceptions, for example one group (the 'green' group) gave quality of life a much higher weighting than the other groups and relatively equal weighting to all other criteria, whilst the 'orange' group focussed highly on prevention, and gave a zero weighting to process.

We performed a sensitivity analysis by substituting mean criteria weightings for each individual group's, to investigate whether there was any effect on the final ranking of the four proposals.

**Figure 3:**
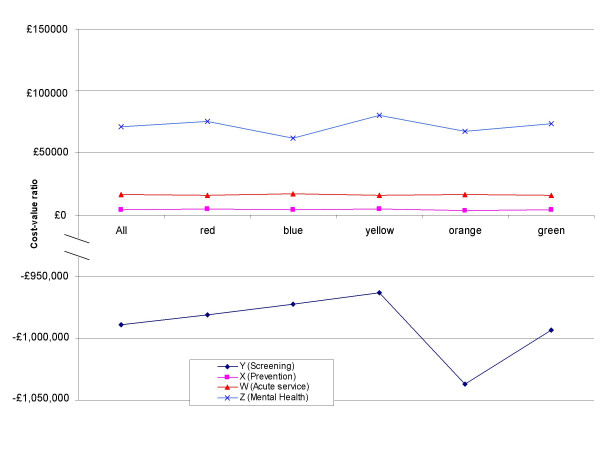
**Sensitivity analysis**. Changes in the cost-value ratios from using mean criteria weights versus each individual group's. The ranking is from lowest to highest cost-value ratio. Note that the lines do not cross meaning the ranked order does not change no matter which set of weights are used. This suggests the ranking is robust to variations in criteria weightings.

Substituting individual group weightings for the average had remarkably little effect on the ranking of the four mock proposals by benefit score alone, with the ordering only changing in the case of the orange group. This group would have ranked proposal X slightly higher than proposal W. This was because the orange group put a great deal of weight on the 'prevention' criterion, and proposal X was itself a programme focussing on prevention of ill health. However, the changes do not affect the cost-value ratios sufficiently to change the ranking (Figure 3).

## Discussion

Similar techniques to prioritising scarce resources have been adopted elsewhere in both the health and non-health sectors. [3-13] Some of these rank options by a weighted benefit score alone [3,5,8], or used no explicit means of prioritising the options. [7,12] The limitation of these methods is that they do not consider the relative value for money of each of the options for service expansion, i.e. the benefit gained for each pound spent. Services are prioritised solely in order of benefit score, without taking into account the resource input required for each unit of benefit / outcome. Therefore overall outcome may not be maximised subject to the available budget.

Our method differed from these by dividing cost into the weighted benefit score to rank in order of 'cost-value'. An alternative method of combining costs and benefits data is the 'prioritisation scoring index' [9]. Here the final ranking is the average of cost and benefit rank. This ensures low cost / high benefit programmes are ranked above high cost / low benefit, but there is a risk of inconsistent results in the middle of the table.

The framework developed most resembles a cost per QALY (Quality Adjusted Life Year) league table [14]. In this case, the outcome of a particular health care programme is measured in QALYs, and ranking in order of incremental cost per QALY gained ensures those at the top of the list, representing best value for money (i.e. greatest gain per pound spent), are funded first. This will maximise the QALYs gained for the population subject to the budget constraint.

However there are limitations with using the QALY approach: QALYs are sensitive to the quality of life tool used to value them [15], and may not capture all dimensions of quality of life relevant to patients. Most importantly, this method assumes that the sole objective of the health care system is to maximise QALYs gained [16]. Wider societal objectives such as access to services, equity and exogenously determined national priorities are not captured by the QALY (although it is postulated that equity weights can be applied to QALYs [17]). Our tool has the advantages of a league table approach (ranking in order of cost-value and therefore ensuring technical efficiency), whilst broadening the measure of benefit to include a comprehensive range of objectives.

A limitation of our method is that is does not consider (in)divisibilities within programmes, and the impact of returns to scale. The implicit assumption is that the 'cost' for a point of benefit is the same, no matter what the scale of the programme. In reality this is unlikely to be so: a scaled down programme may deliver marginally greater benefits, or conversely (due to indivisibilities) may deliver no benefits at all. However, these would be difficult to measure and add a great deal of additional work and complexity to the process.

The criteria used in the framework should be exhaustive and mutually exclusive. There is a risk in our framework that this may not have been so. For example, there may have been overlap between the definitions of 'effectiveness' and 'quality of life'. However, workshop participants were insistent that to them, these were very different concepts (effectiveness relating to clinical measures of response, whilst quality of life was more patient focussed). We felt that for the participants to retain ownership of their framework, it was necessary to allow them to have final say as to the criteria and their definitions.

As stated previously, it is desirable to present results in the form of a 'cost-value' ratio to ensure the most efficient programmes are prioritised. However, this results in a biased ratio as the weighted benefit score does not possess an interval property (the difference between a score of 6 and 8 is not necessarily the same as the difference between a score of 8 and 10). A possible solution is to simply incorporate some measure of resource use (or rather, cost-effectiveness) as a benefit criterion. The ranking is then simply in order of weighted benefit score. This though risks inefficient programmes being prioritised. We decided to use a "cost per patient affected" as the numerator in the cost-value ratio in future implementation: with all costs measured on the per patient level, the cost-value ratio becomes a fairer comparator. This technique has been employed before [4], and partly solves the problem, but this (and the other limitations described) underlines the importance of using the tool as a starting point for discussions rather than the decision itself.

In essence, we have developed a semi-technical method of solving the resource allocation problem. Attempts have been made in the past, most notably in Oregon [18] to make decisions based solely against a set of rational rules. These have run into trouble for both practical (data availability) and conceptual reasons (differing interpretation of the evidence and disagreement as to the purpose of a public healthcare system) [19], and there is concern that overly technical approaches appear more scientific and transparent than they really are [20]. We have emphasised that the rankings generated by our tool should be seen as a starting point from which meaningful discussions can take place to decide priorities, and are not the solution itself. A structured tool such as this provides an essential 'rational' starting point for these discussions (although rationality is bounded within the information set available). Evaluation of its success must be in terms of whether or not a 'better' basket of health services is commissioned as a result of the process compared to without it, not whether the 'perfect' basket could have been commissioned.

Most participants at the workshop felt able to assign a score for each programme against each criterion, and did not feel the framework was lacking any important criteria. However, a number of issues arose during development which required clarification prior to implementation. These were firstly, a practical limitation of testing the tool prior to finalising criteria definitions. The full criteria definitions (Table 1) were not finalised until after the initial workshop. This may have resulted in different interpretations of the same criterion. Secondly, as the focus of the workshops was on developing the benefits framework, the costs of the example proposals were simply presented as net financial impacts, with no greater detail. Thirdly, there is a risk of 'clustering' of scores, and thus a failure to differentiate between programmes. Our sample size in the test run was too small to detect this, but will be monitored as we implement and evaluate the framework.

As a result of these issues and from discussions at further workshops, the finalised criteria definitions list was devised, and that in implementing this tool:

• The three constituencies of patients, managerial / finance and clinicians would be represented by three separate groups who would score proposals independently. Representatives of each of these groups would then meet in a plenary session to agree on final scorings for proposals. This should ensure all 3 groups have equal say in deciding priorities.

• The cost perspective would be societal (including NHS, other agency and patient out of pocket costs), and the time horizon for costs and benefits would be limited to three years.

Limiting the time horizon has the obvious disadvantage of biasing against interventions whose benefits would not be seen within that time span. This would be particularly the case with preventative public health interventions, but it was necessary to compromise between accuracy of data and the risk of underestimating future benefits. During the testing phase of the tool, the preventative programme (X) was ranked highest in cost-value ratio, but only third by benefit score alone. It is therefore not clear whether it would fare so well with a limited time horizon. These issues underline the importance of the PCT Board reviewing the results generated by the process.

## Conclusion

As demands for health care will always outstrip the resources available, prioritisation decisions have to be made in order to maximise the objectives of the PCT subject to the funding available. An explicit, open and transparent prioritisation procedure making use of the evidence base with a clear set of goals and objectives is more defensible than implicit decision making techniques.

There are practical restrictions in implementing any structured, purely technical approach to priority setting (for example data availability, uncertainty and existence of competing goals in the PCT). Consequently any pragmatic scheme will necessarily stray from a theoretic ideal. Furthermore, attempts to correct for subsequent inconsistencies can introduce flaws of their own. Discussion of the results of this framework is therefore essential to confirm priorities.

The benefit scores will be assessed individually by each of three groups (clinical, public, and managerial), before confirmation at a plenary session. Issues arising relating to the knowledge and different experiences of each of these groups and the training and degree of guidance required, e.g. for members of the public group, are being addressed. However, this weakness of having three groups is also a strength: a wide range of individuals from differing of backgrounds contribute to assessing the benefits of proposed programmes of care.

Ultimately, no technical formula can make complex decisions: it can only inform them. Therefore judgement is required: the benefit of this approach is that it forces decision makers to consider why it is they feel a project should be accepted or rejected, and provides a starting point for discussions upon which to make final funding decisions.

Once the process is complete, there will be programmes of care the PCT feels would be of benefit, but fall 'below the line' in the ranked list. The next step is to examine existing expenditure, and consider whether the gains from these new programmes would outweigh losses from potential disinvestments. This is consistent with a Programme Budgeting and Marginal Analysis (PBMA) approach, and this framework would assist in identifying the 'least worst' options from which to disinvest.

The tool is now in use across the PCT and will be evaluated and refined after its first year of operation. By involving a wide constituency in decision making, and explicitly taking into account 'equity' as one of the criteria, we aim to make decision making within the PCT, if not 'fair and equitable' then at least 'fairer' and 'more equitable'.

## List of Abbreviations

NHS National Health Service

PBMA Programme Budgeting and Marginal Analysis

PCT Primary Care Trust

QALY Quality Adjusted Life Year

WBS Weighted Benefit Score

## Competing interests

EW's post is part-funded by West Norfolk PCT. JR is an employee of West Norfolk PCT. RF is director of the Health Economics Support Programme, but does not receive funding from the NHS for this.

## Authors' contributions

All three authors made substantial contributions to the concept and design of the project and led the PCT workshops. EW analysed the data. All authors were involved in drafting and revising this paper, and all give their full approval for this version to be published.
